# Vasopressin 1a Receptor Antagonists for Pathological Aggression in Neurodegenerative and Other CNS Diseases

**DOI:** 10.3390/biomedicines14040889

**Published:** 2026-04-14

**Authors:** Neal G. Simon, Michael J. Brownstein, Karen E. Anderson, Shi-fang Lu, Hilda T. Maibach

**Affiliations:** 1 Azevan Pharmaceuticals, Inc., Bethlehem, PA 18015, USA; mjbrownstein@gmail.com (M.J.B.); sl0d@lehigh.edu (S.-f.L.); 2 Department of Biological Sciences, Lehigh University, Bethlehem, PA 18015, USA; 3 Department of Psychiatry and Neurology, Georgetown University, Washington, DC 20007, USA; kea45@georgetown.edu; 4 Indigo RDD, Potomac, MD 20854, USA; htmaibach@gmail.com

**Keywords:** vasopressin, vasopressin receptors, vasopressin 1a receptor antagonists, aggression, fear circuits, Huntington’s disease, intermittent explosive disorder

## Abstract

**Background:** Neurodegenerative diseases are a major health problem, and the neuropsychiatric symptoms seen in these diseases adversely impact the lives of patients, families, and caregivers. Inappropriate aggressive behavior is a highly disruptive symptom and a leading cause of institutionalization. There are no approved drugs specifically for the treatment of problematic aggression, and the off-label use of antipsychotics has limited benefit with significant side effects and safety risks. This review discusses dysregulated arginine vasopressin (AVP) signaling in fear–threat circuitry as a key driver of inappropriate aggression. Because the AVP 1a receptor (V1aR) is the dominant subtype in the CNS, the selective antagonism of this receptor represents a well-rationalized target for the treatment of aggression across neurodegenerative, psychiatric, and neurodevelopmental disorders. **Objectives:** Our goal was to summarize the basis for using V1aR antagonists as a treatment for irritability and aggressive behavior. We describe its discovery, biosynthesis, receptor pharmacology, and CNS distribution, emphasizing V1aR localization in central fear–threat circuits. Translational evidence from animal studies, pharmacological neuroimaging, and lesion network mapping is presented. These data support the suggestion that heightened vasopressinergic tone biases socioemotional information processing toward negative valence, increasing threat sensitivity and the likelihood of inappropriate aggressive responses. Emerging clinical data support this framework. Highly selective, CNS-penetrant V1aR antagonists reduced aggressive behavior and had an excellent safety profile in phase 2 studies in Huntington’s disease and intermittent explosive disorder, with efficacy signals across caregiver-reported, clinician-rated, and incident-based measures. Furthermore, pharmacological neuroimaging showed that V1aR antagonism normalizes AVP-induced alterations in activity within fear–threat circuitry. **Conclusions and Future Directions:** Preclinical, translational, and clinical findings to date support V1aR antagonism as a promising strategy for treating pathological aggression across disorders. Additional experimental medicine studies and clinical trials are needed to conclusively establish efficacy in various disease populations, and we note the need for improved trial designs and analytical methods as part of the development process.

## 1. Introduction

The social and economic toll of neurodegenerative diseases is devastating. The adverse effects on quality of life for patients and caregivers, escalating direct costs to health care systems, and increased disease prevalence [1,2] demand new approaches to treatment. Recently approved drugs for Alzheimer’s disease and Amyotrophic Lateral Sclerosis, for example, may represent progress in treatment options, but these therapeutics are not cures [3,4,5]. While modestly slowing disease progression and slightly extending lifespan, these drugs are not likely to significantly impact the behavioral and psychiatric symptoms that are a major source of stress for ill persons and their caregivers [6]. In fact, these symptoms are the leading cause of institutionalization as patients become unmanageable at home, and currently used off-label drugs, e.g., antipsychotics, have a range of adverse side effects and carry significant risks [7,8].

The state of treatment for neurodegenerative disorders indicates that until full cures are developed, there is a critical need to develop new treatments for neuropsychiatric symptoms that can benefit quality of life for patients, families, and caregivers. Our focus is on a novel target, the brain vasopressin 1a receptor (V1aR), for the treatment of a prominent and highly problematic behavioral symptom: inappropriate aggressive behavior.

This review covers the history of AVP in the CNS and currently available pharmacological agents, the scientific rationale for targeting V1aR, neurodegenerative and psychiatric conditions where aggression is highly problematic along with currently utilized drugs to manage the behavior, and the need for improving clinical trial design in aggression studies, including measurement challenges, behavioral heterogeneity, and caregiver-reported outcomes. We discuss a mechanistic framework where heightened vasopressinergic tone in fear–threat circuitry biases individuals toward interpreting social cues as threatening, which in turn increases the likelihood of problematic, pathological aggression. V1aR antagonists represent a promising approach for addressing the change in socioemotional cue processing in neurodegenerative diseases and other CNS disorders by normalizing altered information processing.

## 2. Vasopressin and Aggression

In neurodegenerative, psychiatric, and neurodevelopmental disorders, aggressive behavior is counterproductive [6,7]. It is defined by actions that may cause physical, verbal, emotional, and/or psychological harm. In the context of drug development and clinical trials, the heterogeneity in symptom profiles, reporting capabilities, and environmental triggers is a challenge that necessitates tailored approaches to assessment. Reactive aggression is often impulsive and situational, while proactive aggression is calculated and directed. The temporal nature of aggression—whether transient (state) or enduring (trait)—adds to assessment issues.

The neurobiology of aggression is another significant challenge. The behavior is driven by a circuit involving multiple brain regions and mediated by several neurotransmitters including serotonin, norepinephrine, dopamine, glutamate, GABA, corticotropin-releasing factor, opioid peptides, oxytocin, and arginine vasopressin (AVP). Our focus is on the last of these, in part because of the prominent role of AVP in aggression, other social behaviors, and recent clinical studies indicating that this system is a novel and useful therapeutic target for the management of problematic aggression [9,10,11,12,13,14]. Here we discuss the historical background of AVP, its CNS role, and the current state of agonist and antagonist compounds.

### 2.1. AVP in the CNS

A brief review of the history of AVP, including its discovery, biosynthesis, receptor subtypes, and the current status of AVP agonists and antagonists, is provided here.

In 1895, Oliver and Schafer reported that injecting bovine pituitary extracts into dogs increased their blood pressure [15].

Three years later, Howell showed that this so-called pressor effect was driven by the posterior, but not anterior, lobe of the gland [16], and in 1901, Magnus and Schafer found that posterior pituitary extracts reduced the production of urine [17]. On the other hand, when the posterior pituitary (“neurohypophysis”) was removed from animals surgically, they produced abnormally large quantities of urine.

In 1909, two more effects of neurohypophyseal extracts were found. Dale [18] showed that they caused uterine muscle contraction (the oxytocic effect), and Ott and Scott found that they released milk from the mammary glands of lactating animals [19]. After these various effects were discovered, a number of workers tried to isolate the factor(s) responsible for them. Depending on the biological assays used, some concluded that a single factor drove them all; others believed that two or more factors were involved. Abel was able to concentrate the pressor factor but never purified it. By 1928, Kamm and colleagues succeeded in separating the four activities into two high-potency fractions [20]. One contained most of the pressor and antidiuretic activities, and the other had the oxytocic and milk-releasing factors. Over time, the first of these was attributed to “vasopressin” and the second to “oxytocin”.

Du Vigneaud and coworkers [21] grew interested in these hormones after hearing about the success that Banting and Best had had in treating diabetes mellitus with insulin preparations. Du Vigneaud was able to improve the method that Abel had used to crystallize insulin and spent a year in Bergmann’s laboratory learning how to synthesize peptides. Then he studied Kamm’s posterior pituitary extracts, concluding, among other things, that the sulfur present in the oxytocin and vasopressin fractions was contributed by cysteine. He ultimately used electrophoresis to resolve and further purify the fractions of interest. During World War II, du Vigneaud worked on methods for producing penicillin and mastered additional methods that proved useful in purifying peptides. After the war, he returned to work on oxytocin and vasopressin and managed to make milligram quantities. Focusing on oxytocin initially, he used a method developed by Stein and Moore to identify the amino acids that made up the hormone and a technique like Sanger’s to determine their order. Finally, he developed a method for synthesizing the peptide and described its properties in 1953. The natural and synthetic peptides were identical in every physical, chemical, and biological assay. In fact, the synthetic peptide induced labor in women who were about to give birth when it was given intravenously, and it quickly released milk from the mammary glands of women who were nursing. Shortly after they characterized oxytocin, du Vigneaud and his coworkers also sequenced and synthesized vasopressin [22].

### 2.2. Biosynthesis of AVP

When neurophysins were discovered by Archer et al. [23], they were believed to be inactive fragments of putative vasopressin and oxytocin precursor proteins and to function as carriers for peptides. (They are still thought to play an essential role in carrying and protecting peptides during axonal transport). Taking advantage of the fact that vasopressin, oxytocin, and neurophysins are rich in cysteine, Gainer et al. pulse-labeled the precursors with S35-cysteine [24]. This allowed them to study their synthesis and processing in the cell bodies, axons, and terminals of magnocellular neurons in the supraoptic and paraventricular nuclei, but it was not until Richter et al. cloned cDNAs encoding preprovasopressin and preprooxytocin that we had a detailed picture of these gene products [25]. Preprovasopressin is a 164-amino-acid protein encoded by the 2.5 kb AVP gene which, in humans, is in chromosome region 20p13. It comprises a signal peptide, AVP, neurophysin II, and a glycopeptide called copeptin. The oxytocin precursor lacks a copeptin analog. Provasopressin is produced when the signal peptide is removed from preprovasopressin and a carbohydrate chain is added to the glycopeptide. Then the precursor is cut into three parts, extra basic amino acids are pruned off, and vasopressin undergoes alpha-amidation at its C-terminus.

### 2.3. AVP in the Central Nervous System

Ernst and Berta Scharer suggested in the 1920s that “neurosecretory” agents might be made by brain cells and released into the bloodstream [26], but it was not until the 1970s that methods for visualizing molecules like peptides were routinely used by neuroanatomists. Before that, people who were interested in the distribution of peptides in the brain used radioimmunoassay to measure their levels in tissue samples. In this way, workers in the field discovered that “hypothalamic hormones” like GnRH and somatostatin were likely to be made by cells outside of the hypothalamus as well as ones that are in it [27]. This seemed to be true of vasopressin, which Glick and Brownstein found in most regions of the brain [28]. They detected relatively high levels of the peptide in the amygdala, septum, thalamus, and striatum, confirming earlier immunohistochemical studies published by Weindl and Sofroniew [29] who found vasopressin in parvocellular neurons in the paraventricular nuclei, suprachiasmatic nuclei, triangular nucleus of the septum, and organum vasculosum of the lamina terminalis.

### 2.4. AVP Receptors

By the beginning of the 1990s, workers in the field realized that there were three vasopressin receptors and one (related) oxytocin receptor. There were two V1 receptors, V1a (vascular/hepatic) and V1b (anterior pituitary), that were understood to act through phosphatidylinositol hydrolysis to mobilize intracellular Ca^2+^. The oxytocin receptor had a similar mechanism of action, and a single V2 receptor was known to activate adenylate cyclase in the kidney, producing the antidiuretic effect of vasopressin. While a good deal was known about these receptors, their structures and detailed mechanisms of actions remained a mystery. In no small part, this was because G-protein-coupled receptor proteins are relatively non-abundant, as are the mRNAs that encode them. Brownstein and colleagues finally succeeded in using expression cloning to isolate rat V1a receptor and human oxytocin receptor cDNAs [30,31]. Based on their structures, they were also able to isolate human V2 and rat V1b receptor cDNAs. Expressing these cDNAs in Chinese hamster ovary or COS-7 cells allowed them and others to examine their properties in detail, determine where the receptors were expressed in the body, and screen synthetic peptide and small molecule libraries for receptor-specific agonists and antagonists.

Currently available V1a and V1b receptor agonists and antagonists are shown below (Table 1 and Table 2) The V1a receptor is found in many tissues in the periphery: the liver, vascular smooth muscle, heart, platelets, adrenal gland, testes, and urinary bladder. In the brain, it is present in the cerebral cortex, hippocampus, hypothalamus, olfactory bulb, striatum, and brainstem [32,33]. The highest V1b receptor levels have been measured in the anterior pituitary (in corticotropes) and in pancreatic islet beta cells. The V1b receptor is also present in the kidney, thymus, heart, lung, spleen, uterus, adrenal gland, and breast. The V2 receptor is on the principal cells of the renal collecting duct, and as noted earlier, it mediates the antidiuretic effect of vasopressin. V2 receptors are reportedly present in the inner ear, where they may regulate endolymphatic sac pressure. Defects in the V2 gene result in nephrogenic diabetes insipidus [34], while central diabetes insipidus (CDI) results from the failure of the pituitary to store and release enough vasopressin. CDI can be caused by tumors, head injuries, surgery, or genetic mutations. Other mutations lead to the constitutive activation of the V2 receptor and hyponatremia (nephrogenic syndrome of inappropriate antidiuresis, NSIAD; [35]).

## 3. Vasopressin, the V1a Receptor, and Inappropriate Aggression

Arginine vasopressin significantly contributes to the regulation of a broad range of social behaviors, including dominance and aggression [9,11]. AVP neurons originating primarily from the paraventricular (PVN) and supraoptic (SON) nuclei project extensively to circuits involved in emotional evaluation and both offensive and defensive aggressive behaviors [35,36,37]. These effects are primarily mediated through V1aR, which is extensively expressed in the brain in non-human primates and humans [32,38,39].

In human clinical populations, several lines of evidence implicate increased vasopressinergic tone in pathological aggression. AVP concentrations in the cerebrospinal fluid were positively correlated with life histories of impulsive aggression [40]. Neuroimaging and other studies demonstrated that intranasal AVP alters the circuitry involved in fear/threat responses and the interpretation of socioemotional stimuli [35,36,37,41,42]. Pharmacological findings demonstrated that V1aR antagonists reduced aggressive behavior [14].

### 3.1. Neuroanatomical Substrates

V1aR is enriched in regions that are core components of fear/threat circuitry. There is an abundant body of literature on rodent models demonstrating that the amygdala, several hypothalamic nuclei, the bed nucleus of the stria terminalis (BNST), the lateral septum, and multiple cortical regions are involved in these responses (e.g., [11,43]). In addition to collectively regulating fear/threat processing, these regions, and AVP signaling, are strongly involved in social recognition and arousal. A degree of caution is needed, however, because our interest is in inappropriate aggression in neurodegenerative (and psychiatric) indications, while much of the aforementioned work relied on typical, species-specific aggressive behavior in rodents that is unrelated to brain disease. In addition, we are well aware of the fact, as noted earlier, that AVP is one part of a highly complex signaling system mediating aggressive responses. Despite this caution, there is a long-recognized, strong rationale from a clinical and translational perspective for the potential utility of V1aR antagonists [14,44,45,46,47] as a means to treat inappropriate aggression, which is the focus of our discussion. It should also be mentioned that V1aR antagonists are potential therapeutics for several stress-related and neurodevelopmental disorders, including anxiety, depression, PTSD, and problematic behaviors in autism spectrum disorder (ASD; [48,49,50,51]).

Underpinning the use of V1aR antagonists as a therapeutic intervention for inappropriate aggression is the demonstrated effect of AVP on the fear/threat circuit, the ability of V1a antagonist treatment to reduce the heightened sensitivity to negatively valenced stimuli induced by AVP, and the decreased aggression seen in two disorders, Huntington’s disease and intermittent explosive disorder [14,37]. Neuroimaging studies in humans show that the circuit broadly incorporates the temporoparietal junction (TPJ), frontal cortex regions, anterior cingulate cortex, insula, hippocampus, striatum, and the amygdala [37,52]. Intranasal AVP administration alters BOLD signaling in several of these regions, and V1aR antagonist treatment with SRX246 normalizes the changes [37,41,42].

The potential role of the TPJ merits attention as a significant contributor to pathological aggression in neurodegenerative and psychiatric disorders within the frame of an altered information processing hypothesis. The right TPJ (rTPJ) in particular plays a central role in the evaluation of sensory, cognitive, and affective information that determines emotional valence [53,54,55]. In regard to inappropriate aggression, the rTPJ promotes exaggerated negative emotional valence, likely by weakening the cortical regulation of threat processing, primarily in the amygdala. The effect likely involves the disruption of the role of the TPJ in default mode and social cognition networks, which is correlated with a shift toward negative bias in the interpretation of social stimuli [56,57]. Importantly, dysregulated AVP signaling through V1aR can function as a gain mechanism for social salience and threat detection [12,37]. AVP–V1aR signaling enhances amygdala responsivity and modulates extended limbic circuits, including components of the fear/threat circuit (e.g., hypothalamic and prefrontal regions), an effect that can increase sensitivity to threatening cues. Vasopressin modulates activity within the TPJ during social recognition tasks, and V1aR antagonist administration attenuates threat-related amygdala responses through effects in the dorsolateral and anterior cingulate cortices, which are involved in social–emotional evaluation [37,41,42].

### 3.2. Network Mapping

The fear–threat circuit encompasses multiple subcortical and cortical regions and inputs. Behavioral output reflects the integration of salience, threat detection signals, and their interpretation. Altered signaling or damage impacting core nodes or their interconnections thus may bias an evaluation toward threat and increase the likelihood of pathological aggression. This view is strongly supported by a recent lesion network mapping study [58] that examined violent and aggressive behavior in individuals with focal brain lesions. Peng and colleagues demonstrated that lesion sites associated with aggression were functionally connected to a common distributed network that included the temporoparietal junction, medial and lateral prefrontal cortices, posterior cingulate cortex, and lateral temporal regions, with negative connectivity to limbic structures, including the amygdala and hippocampus. These findings indicated that aggression-related behavior emerged when lesions disrupt cortical systems that normally exert regulatory control over limbic threat processing, independent of the anatomical location of the damage. Importantly, this network extensively overlaps with regions implicated in social cognition and emotional evaluation, which are often compromised early in the development of neurodegenerative disease and contribute to pathological aggression. Other human imaging work suggests that attenuated prefrontal control and heightened amygdala reactivity are associated with aggressive and disruptive behaviors [59,60].

Within this framework, AVP signaling through V1aR seems to be an important driver of aggression. Increased vasopressinergic tone can function either to amplify the negative valence of social stimuli and increase the likelihood of an aggressive response as described above or serve as a direct driver of the behavior through disinhibited regulatory pathways. Human pharmacological neuroimaging studies support this model, demonstrating that AVP administration enhances reactivity to emotional stimuli, while V1aR antagonism attenuates these limbic responses and normalizes activity in cortical regions involved in social–emotional stimulus processing [37]. When considered alongside Peng et al. [58], these results suggest that AVP/V1aR signaling may exert disproportionate influence when right temporoparietal junction–prefrontal cortex and consequently fear–threat networks are compromised. The effect is a shift in emotional appraisal toward negative valence and increasing susceptibility to display aggression.

### 3.3. Clinical Implications

It seems reasonable to support the concept that pathological aggression in neurodegenerative and psychiatric disorders reflects an interaction between the structural or functional disruption of regulatory networks and increased sensitivity in limbic threat detection systems. In these disorders, where network integrity progressively degrades (e.g., [52]), increased AVP activity through V1aR may contribute to exaggerated threat perception, anger, and aggressive behavior. The antagonism of V1aR is thus well-rationalized as a potential therapeutic intervention. In accord with this view, V1aR antagonists have shown promise in reducing threat hypersensitivity and mitigating inappropriate aggression. Potential indications, in addition to neurodegenerative diseases, include PTSD, traumatic brain injury, autism spectrum disorder, intermittent explosive disorder, and other impulse control disorders.

## 4. Vasopressin and Aggression: Clinical Considerations

Inappropriate aggression is a pervasive issue in psychiatry and neurology—in addition to neurodegenerative diseases, it is also seen in patients who have stroke, traumatic brain injury, and intellectual disabilities such as Fragile X syndrome. It is a core feature in intermittent explosive disorder and is often seen in other psychiatric conditions, such as depression, anxiety, and bipolar disorder.

The adverse impact of aggression can be far-reaching and severe, including rejection by families, injuries to patients and caregivers, involvement with law enforcement, and institutionalization. Remarkably, there are no approved medications specifically for the management of this behavior in neurodegenerative diseases. Many of the off-label medications used to treat aggression have side effects such as sedation, the worsening of cognitive symptoms, and increased fall risk [8]. Antipsychotic medications, which are often used to control aggression, have an FDA black box warning for morbidity and mortality in elders. Two antipsychotics, risperidone and aripiprazole, are approved for the treatment of irritability and aggression in autism [61,62]. These factors reinforce the need for symptomatic treatment with minimal side effects, a need that may become even more pressing if disease-modifying treatments become available. This could be the case with recent approvals in Alzheimer’s disease, which could result in affected individuals living longer with conditions that may cause aggression.

### 4.1. V1aR Antagonists in Recent Clinical Trials

Non-peptide V1a antagonists were introduced by Sanofi [63] as potential therapeutics across a range of peripheral indications. However, they did not cross the blood–brain barrier (BBB) with oral administration, which precluded their use in CNS indications. These early V1a antagonists were not developed clinically.

Balovaptan (Roche) and SRX246 (Azevan Pharmaceuticals), two highly selective, highly specific, orally bioavailable V1a antagonists, have recently been in multiple clinical trials: Balovaptan was studied as a potential treatment for abnormal behavior in ASD. with a primary emphasis on improving social communication. Improvements in this aspect of behavioral function could decrease aggressive behaviors in autism spectrum disorder. The most common side effects of Balovaptan in clinical trials were headache, nausea, diarrhea, and upper respiratory tract infection. A phase 2 trial showed mixed results, with some scales demonstrating improvements in social communication and others showing no significant effect [50]. A phase 3, randomized, placebo-controlled, double-blind trial (V1aduct) did not meet its primary endpoint of improving social communication in adults with ASD [64,65]. SRX246 has been in phase 2 trials for the treatment of intermittent explosive disorder and irritability/aggression in Huntington’s disease [14]. It also was tested as a treatment for PTSD, although aggression was not an emphasis of that trial [49].

### 4.2. Specific Conditions

#### 4.2.1. Huntington’s Disease

HD is an inherited disease resulting from the expansion of a trinucleotide (CAG) repeat in the coding region of the huntingtin gene. Symptoms include behavioral, cognitive, and motor problems such as chorea, bradykinesia, gait impairment, dysarthria, and dysphagia. Disease onset is typically between age 35 and 44 but may start earlier or later. HD is a rare disease, affecting 4.1–5.2 people per 100,000, and is more common in Caucasian populations.

Psychiatric symptoms, including aggression, are common in HD and are quite distressing. Aggression prevalence is between 22 and 66 percent depending on the population studied and assessments used [66]. It may be more common in men with HD and is seen more often in those who experience frequent falls and have obsessive and compulsive behaviors and suicidal ideation. The behavior, as noted above, adversely impacts daily life and often results in institutionalization [67,68]. Problematic aggression is associated with an earlier age of onset. It may occur in patients who are younger and stronger, posing more safety threats to others [69]. In spite of this, it has received little attention as a therapeutic target. Currently, antipsychotics, mood stabilizers, and antidepressants are used off-label to treat behavioral problems, but they have limited effect on aggression except at high doses, where side effects are problematic [70]. As noted above, the V1a receptor antagonist, SRX246, was studied for irritability and aggression in HD in an exploratory phase 2 trial, Safety, Tolerability, and Activity of SRX246 in Irritable HD patients [14,71]. SRX246 was safe and well tolerated. Endpoints included the Cohen-Mansfield Agitation Inventory (CMAI), eDiary patient and caregiver reports, and caregiver burden questionnaires. The exploratory analyses showed that SRX246 reduced aggression. It also demonstrated that observable behaviors, like aggressive acts (yelling, hitting), were easier to measure than internal states of irritability.

#### 4.2.2. Intermittent Explosive Disorder (IED)

Defined by recurrent, problematic, impulsive aggression, IED is a common behavioral disorder for which there is an unmet need for pharmacological and/or psychological interventions. Aggressive outbursts in IED have a rapid onset and little or no prodromal period. Episodes are typically less than 30 min and involve verbal assault, destructive and non-destructive property assault, or injurious or non-injurious physical assault. Aggressive outbursts most commonly occur in response to a minor provocation by a close intimate or associate. It is often comorbid with other psychiatric disorders such as depression, anxiety, bipolar disorder, conduct disorder, oppositional defiant disorder, post-traumatic stress disorder, substance use disorder, antisocial personality disorder, and borderline personality disorder.

There are no FDA-approved treatments for IED. The off-label use of medications such as selective serotonin reuptake inhibitors (SSRIs), mood stabilizers, and antipsychotics is common. Systematic studies of efficacy are limited. There appeared to be a decrease in impulsive aggression in a double-blind, placebo-controlled, clinical trial of fluoxetine in patients with personality disorders who also suffered from IED [72,73]. A decrease in impulsive aggression in patients with Cluster B personality disorder with IED was also seen following treatment with divalproex [74] and oxcarbamazine but not leviracetam [75,76]. In an exploratory phase 2 study with SRX246, the drug was well tolerated, and the adverse events seen in patients who completed the 8-week regimen were mild, transient, and not dose-dependent. There were positive trends in the data, especially from instruments that relied on Patient-Reported Outcomes. The scales that proved to be the most useful were STAXI, OAS-M (weekly, numerical reporting of aggressive events), Aggression Outburst Disability Scale (lost and unproductive days due to outbursts), and the BAI. The STAXI results suggested that SRX246-treated subjects may feel less angry than placebo-treated controls; the weekly reports of aggressive events in the OAS-M supported this conclusion. SRX246-treated patients had fewer aggressive outbursts than those who were given placebo. At the end of the trial, subjects in the active arm reported a significant decrease in lost work or school days. This improvement may have been related to a reduction in anxiety relative to placebo-treated controls based on lower BAI scores with SRX246 treatment. However, the statistical power of the trial was impacted by the loss of subjects, failure of some participants to take the study drug, and, in some cases, site performance (unpublished data, Azevan). Further testing with study design improvements and steps to assure compliance seems warranted.

#### 4.2.3. Fragile X Syndrome

Fragile X syndrome (FXS) is a genetic disorder caused by a mutation on the X chromosome affecting the FMR1 gene that primarily impacts development, causing a distinctive phenotype (long, narrow face; large ears; prominent jaw; high arched palate; hyperextensible fingers; flat feet; and enlarged testicles in males after puberty) along with intellectual disability and social and behavioral deficits. It is considered the most common inherited cause of intellectual disability. FXS is more common in males, affecting approximately 1 in 4000 compared to 1 in 6000 to 8000 females with an overall prevalence of 1 in 7000 individuals.

Aggression is common in individuals with FXS and may be associated with sensory overload, anxiety, and impulse control problems. Aggression was the most severe reported behavior problem by the parents of children with FXS in one study, where the daily prevalence for aggression was between 20% and 40% [77]. In the same cohort, 25% of caregivers reported this behavior as a safety threat. In a study of FXS males aged 6–47 years, 21.5% exhibited persistent aggressive behavior, and higher impulsivity scores were associated with an increased probability of aggression [78]. Aggression was also found to be the most prevalent behavioral problem in this group.

The treatment of aggression in FXS relies mainly on antipsychotics, particularly risperidone and aripiprazole. Both may show some efficacy [79,80,81]. As noted in a recent review, there is a dearth of systematically collected information on the actual efficacy of these agents when used in FXS, and there also is a substantial risk of side effects [82]. This has led to efforts to deprescribe antipsychotics to FXS patients [83]. Antidepressants are commonly prescribed to individuals with FXS for numerous behaviors, but they may cause paradoxical disinhibition, limiting usefulness for aggression [81]. Mood stabilizers, particularly lithium [84], may be helpful for aggressive behaviors in FXS, but again, not all studies show benefit [84,85]. Side effects from mood stabilizers can also be a limiting factor. The use of benzodiazepines and buspirone is reported, but there is little systematic research on these agents. Paradoxical disinhibition from benzodiazepines and the potential for tolerance leading to dose escalation and the risk of addiction have resulted in limited use in FXS.

### 4.3. Other Psychiatric Indications

Anxiety, which consists of excessive fear or worry, can be associated with aggression [86,87]. Deficits in social communication, seen in many psychiatric conditions, can lead to the dysregulation of emotions, resulting in aggression [88]. Anxiety disorders are typically characterized by the avoidance of social situations, but anxiety disorder patients may demonstrate intense anger and aggression [89,90]. Individuals with anxiety disorders can have difficulty recognizing and processing negative emotional states, such as feelings of rejection by others. This suppression of uncomfortable feelings has the unfortunate effect of increasing physiological arousal, which might lead to aggressive outbursts [91]. Clinical work has shown increases in aggression among individuals with anxiety disorders, a high comorbidity of anxiety disorders with antisocial behavior and aggression, and poor treatment outcomes for individuals with anxiety who also have anger problems [90,92].

Prevalence data are not readily available for the rates of aggression in those with anxiety symptoms. An analysis of data from the National Comorbidity Survey Replication (N = 9282) and Adolescent Supplement (N = 9632), representing over 18,000 individuals, examined the comorbidities of several psychiatric conditions, including anxiety and aggression [93]. They found that adolescents with a lifetime anxiety disorder had a higher prevalence of lifetime anger attacks (68.5%) and IED (22.9%) than those without a lifetime anxiety disorder (48.6% and 7.8%, respectively). This was particularly true of social phobia and panic disorders. Similarly elevated rates were seen in adults.

There has been limited work in humans to date with vasopressin antagonists for the treatment of anxiety, and no studies have focused directly on the topic of aggression associated with anxiety. The novel V1a receptor antagonist, SRX246, decreased anxiety-potentiated startle in a study of 36 healthy volunteers [48]. Because anxiety-potentiated startle is elevated in anxiety disorders, V1aR antagonists may be a potential therapeutic agent for anxiety. This view is at least partially supported by the previously mentioned finding in intermittent explosive disorder, where SRX246 treatment lowered anxiety as assessed by the Beck Anxiety Inventory.

Depression, which consists of a low mood along with neurovegetative symptoms such as change in appetite, disordered sleep, negative thoughts, and impaired cognition, is associated with aggression [94,95]. There are few studies looking at the prevalence of aggression in individuals with major depression. One large study examined the prevalence and correlates of aggressive outbursts among adults with primary Major Depressive Disorder (MDD, *n* = 2539) from the Collaborative Psychiatric Epidemiological Surveys [96]. The prevalence estimate was almost 60% among adults with MDD for any aggressive outbursts. MDD was associated with aggressive outbursts independent of other psychiatric diagnoses. Aggressive outbursts in MDD were associated with greater severity and an earlier age of onset for MDD. Other positive associations were reported between the frequency of aggressive outbursts and depressive symptoms, including weight/appetite change, fatigue, and recurrent thoughts of death. Having aggressive outbursts increased the risk of more severe functional impairment, suicidal ideation, suicide plan, and suicide attempts.

## 5. Defining and Measuring Aggression Across Disorders: Toward a Multi-Instrument Framework

Aggression is commonly defined as verbal or physical behavior directed toward another individual with the intent to cause harm. In clinical contexts, this definition is inadequate; a measurement model of aggression that includes multidimensional phenomena shaped by neurocognitive capacity, emotional regulation, impulse control, environmental context, and intentionality is needed [97,98,99,100].

In neurodegenerative disorders such as Alzheimer’s disease (AD), aggressive behavior often reflects agitation, resistance to care, or fear-based responses arising from confusion, pain, or unmet needs rather than deliberate hostility [97,98,99]. In Huntington’s disease (HD), irritability and disinhibition frequently precede more overt verbal or physical aggression and are closely linked to disease progression and frontostriatal dysfunction [101,102,103]. In contrast, aggression in FXS and other neurodevelopmental disorders often manifests as impulsive, sensory-driven, or self-directed behavior related to anxiety and impaired emotional regulation [104,105,106]. Intermittent explosive disorder (IED) represents a distinct phenotype characterized by recurrent, impulsive aggressive outbursts that are grossly disproportionate to provocation and occur in the absence of progressive neurodegeneration [107,108,109,110,111].

These distinctions have critical implications for measurement when testing for drug effects in specific indications. The implications for successful therapeutic development are obvious—instruments optimized for one population may fail to capture clinically meaningful aggression in another, leading to misclassification, reduced sensitivity to change, and the limited interpretability of trial outcomes.

### 5.1. Aggression as a Multidimensional and Temporally Dynamic Construct

As noted previously, aggression spans multiple behavioral domains. Beyond behavioral distinctions, aggression also varies along additional dimensions such as frequency, severity, disruptiveness, and contextual triggers. Importantly, aggression may reflect either transient state phenomena (e.g., episodic outbursts) or stable trait-like dispositions (e.g., chronic irritability or hostility), and these dimensions are not interchangeable [107,108,109,110,111].

Temporal dynamics further complicate assessment. In neurodegenerative disorders, aggression may fluctuate with disease stage, environmental stressors, or caregiver interactions. Intermittent explosive disorder provides a contrasting example, where outbursts are typically brief, episodic, and clustered in response to minor provocations [107,108,109]. Capturing these dynamics is essential in clinical trials, where endpoints must reflect both short-term behavioral change and longer-term functional impact.

### 5.2. Assessment Instruments and Scoring Considerations

A wide array of instruments has been developed to assess aggression, differing in respondent type (self-report, informant-rated, clinician-rated), recall window, scoring structure, and administration format [97,98,99,109,110,111]; see Table 3 and Table 4. The Neuropsychiatric Inventory (NPI), for example, relies on caregiver reports and combines frequency and severity ratings over a four-week recall period, making it well suited for global behavioral assessment in dementia but vulnerable to recall bias [97]. The Cohen-Mansfield Agitation Inventory (CMAI) emphasizes frequency over a shorter recall window and is particularly sensitive to repetitive agitation and care resistance behaviors; however, it captures a mixed agitation–aggression construct rather than aggression alone [98].

Clinician-rated instruments such as the Overt Aggression Scale-Modified (OAS-M) provide high temporal resolution by documenting weekly aggressive incidents across defined domains, but they may be influenced by observer expectations and variability in clinical documentation [107,108]. Disease-specific instruments, such as the Problem Behaviors Assessment for Huntington’s disease (PBA-HD), integrate frequency and severity ratings, as well as capturing irritability and quickly changing emotional states central to HD-related aggression, albeit at the cost of greater administrative complexity [101,102,103].

Self-report instruments, including the Buss–Perry Aggression Questionnaire (BPAQ) and the Life History of Aggression (LHA), efficiently assess trait-level aggression and historical patterns, but they are of limited value in populations with impaired self-insight, cognitive decline, or restricted communicative capacity [109,110,111].

### 5.3. Bias and Limitations in Aggression Measurement

Aggression assessments in clinical trials are unfortunately subject to systematic bias introduced by study design, measurement, conduct, and analysis that “generalizes” across groups. As mentioned above, informant-rated tools are particularly vulnerable to recall bias and emotional framing, especially when recall windows extend beyond one to two weeks [97,98]. Long-term caregivers may become desensitized to aggressive behavior, leading to underreporting, whereas acute stress may inflate severity ratings. Repeated assessments within the trial may heighten rater focus on specific behaviors, introducing systematic shifts that are independent of true behavior change. Patient- or caregiver-reported measures are also susceptible to social desirability bias, while clinician ratings may reflect diagnostic anchoring or incomplete observation [107,111].

To mitigate systematic under- or overreporting, informant-rated outcomes should be paired with clinician-rated or incident-based measures [117]. Discrepancies across informants (patient, study partner/caregiver, observer, clinician) should be anticipated and modeled analytically rather than interpreted as measurement failure.

### 5.4. Open-Ended Descriptions: Contextual Depth Versus Standardization

Several aggression instruments incorporate open-ended narrative components, including the Behavioral Pathology in Alzheimer’s Disease Rating Scale (BEHAVE-AD) and narrative fields within the OAS-M [99,107,109]. These qualitative descriptions provide valuable contextual information regarding triggers, environmental contributors, and emotional antecedents [118].

However, narrative data are difficult to standardize and integrate quantitatively. Variability in language, detail, and interpretive framing limits cross-participant comparability. When systematically coded, qualitative data can augment quantitative outcomes. However, we believe that these data should be viewed as complementary rather than primary endpoints.

### 5.5. Disease-Specific Validation and Limits of Harmonization

Instruments validated in one population may perform poorly in another. Tools developed for institutionalized dementia populations may not generalize to outpatient neurodevelopmental cohorts and vice versa. Disease-specific validation is therefore essential to ensure construct validity, sensitivity to change, and clinical interpretability.

Attempts to harmonize aggression measures across disorders—such as converting scores to standardized metrics or pooling endpoints—must be approached with caution. Trait-based instruments (e.g., BPAQ, LHA) and incident-based measures (e.g., OAS-M) should not be combined without the explicit modeling of temporal scope and construct differences.

### 5.6. The Rationale for a Multi-Instrument Strategy (Including the Role of Factor Analysis)

Given these limitations, a multi-instrument framework offers a pragmatic solution. Pairing high-frequency incident tracking (e.g., OAS-M) with broader contextual or trait-based measures (e.g., BPAQ, LHA, CMAI-Relatives, PBA-HD) allows investigators to capture both temporal dynamics and overall behavioral burden. The inclusion of disruptiveness or functional impact ratings further distinguishes frequent but low-impact behaviors from infrequent yet clinically consequential events.

Multivariate analytic approaches—including regression modeling and correlation analyses—can identify which instruments uniquely predict functional outcomes or treatment response while minimizing redundancy. Factor analytic methods are sometimes proposed as tools for dimensionality reduction; however, their utility is constrained. Within a single diagnostic population, factor analysis may help identify separable dimensions when applied to instruments capturing overlapping constructs. By contrast, cross-instrument or cross-condition factor analysis is unlikely to yield stable or clinically meaningful factors because aggression scales differ fundamentally in item content, time horizon, and construct intent. Pooling incident-based and trait-based measures violates the assumptions of temporal and construct equivalence and risks producing statistical artifacts rather than interpretable dimensions.

Accordingly, factor analysis should be viewed as a supplementary, population-specific tool, not a unifying solution for harmonizing aggression outcomes across disorders. Multi-instrument strategies that preserve the distinct informational value of individual measures are more likely to yield clinically interpretable and regulatory-relevant endpoints.

### 5.7. Assessment Conclusions

Aggression is a complex, context-dependent construct that cannot be adequately captured by a single assessment tool across disorders. In neurodegenerative, neurodevelopmental, and psychiatric populations, meaningful measurement requires alignment between instrument design, disease phenomenology, and trial objectives. Multi-instrument strategies—validated within specific populations and analyzed with attention to bias, temporal dynamics, and construct boundaries—provide the most robust foundation for clinical trials and patient-focused drug development.

## 6. General Conclusions

Several lines of evidence support V1aR hyperactivation as a central mechanism in inappropriate, pathological aggression. Animal, human imaging, molecular, and lesion network data point to V1aR as a viable therapeutic target. The potential utility of V1aR antagonists across disorders lies in the perturbation of vasopressin signaling at V1aR as a common underlying mechanism. The increased vasopressinergic tone can occur as a result of disinhibition in fear–threat circuitry, elevated vasopressin secretion, or enhanced receptor sensitivity. V1aR antagonism should be clinically efficacious regardless of the basis for enhanced CNS vasopressin activity. Effective clinical development, however, will require rigorously designed clinical trials in carefully defined populations within neurodegenerative (and other) diseases—improved measurement strategies will be essential to advancing new therapeutics. These steps are essential for advancing new drugs like V1a antagonists as therapeutic interventions to reduce pathological aggression and improve quality of life for persons with these disorders and their families. Another aspect in developing V1a antagonists will be the characterization of changes in the brain produced by drugs that mediate clinical benefit. Our working hypothesis, supported by translational imaging and additional results from our group and others, focuses on increased vasopressin sensitivity that leads to dysregulated fear/threat circuit function, which in turn increases the probability of inappropriate, pathological aggressive responses. While there are open questions about neurological changes that result in AVP-mediated enhanced fear/threat sensitivity, addressing these would more fully elaborate the mechanism of action mediating the clinical utility of V1aR antagonism.

## Figures and Tables

**Table 1:** Vasopressin and oxytocin receptor agonists.

Ligand	Origin/Type	Key Structural Features	Receptor Activity	Clinical Use
Arginine vasopressin (AVP)	Endogenous (human)	Cys1–Cys6 disulfide; amidated C-terminus	V1a/V1b/V2 agonist	Water balance, vasoconstriction
Lysine vasopressin (LVP)	Endogenous (porcine)	Lys at position 8	AVP-like	Parent of terlipressin
Oxytocin	Endogenous	Ile3, Leu8 substitutions	OXTR; cross-activation of V1a/V2	Parturition, lactation, social behavior
Desmopressin (dDAVP)	Synthetic analog	Mpa1, D-Arg8	Selective V2 agonist	Diabetes insipidus, bleeding disorders
Terlipressin	Synthetic prodrug	N-terminal triglycine	Converted to LVP	Variceal bleeding, HRS
Selepressin	Synthetic peptide	Backbone modifications	Selective V1a agonist	Septic shock (discontinued)
Felypressin	Synthetic analog	Phe at position 2	V1a agonist	Dental vasoconstriction
Ornipressin	Synthetic analog	Ornithine at position 8	V1a agonist	Hemostasis (toxic)

**Table 2:** Orally and intravenously active non-peptide vasopressin receptor antagonists are known as vaptans.

Compound	Receptor Selectivity	Route	CNS Penetration	Clinical Status/Indication
Tolvaptan	V2	Oral	No	ADPKD; hyponatremia
Conivaptan	V1a/V2	IV	Limited	Acute hyponatremia
Lixivaptan	V2	Oral	No	Hyponatremia, ADPKD (not approved)
Satavaptan	V2	Oral	No	Cirrhosis ascites (ineffective)
Nelivaptan	V1b	Oral	Yes	Anxiety, depression (halted)
Balovaptan	V1a	Oral	Yes	ASD, PTSD (ineffective)
Relcovaptan	V1a	Oral	Limited	Raynaud’s (discontinued)
Mozavaptan	V2	Oral	No	SIADH (Japan)
Serevaptan (SRX246)	V1a	Oral	Yes	Aggression (in development)
SRX251	V1a-related	Oral	Yes	TBI (preclinical)

**Table 3:** Behavioral instruments used to assess aggression, agitation, and related constructs across neurodegenerative, neurodevelopmental, and psychiatric populations.

Instrument	Construct Domain	Original Target Population	Validated Population(s)	Key Strengths	Key Limitations/Notes
ABC-C [104]	Aggression/irritability in DD	DD, FXS, autism	ID/DD	Strong DD/FXS validity	Not adult/neurotypical-specific
BPI [106]	Aggressive/destructive behaviors	ID/DD	ID/DD	Explicit aggression coverage	Caregiver burden
BEHAVE-AD [99]	Dementia behavioral pathology	Alzheimer’s disease	AD dementia	AD-specific coverage	Not impulsive aggression-focused
CMAI [98]	Agitation/aggression	Elderly dementia	Institutional dementia	Sensitive to change	Non-aggressive agitation included
RAGE [112]	Overt aggression (elderly)	Institutional elderly	Dementia inpatient	Captures staff burden	Setting-dependent
NPI [97]	Neuropsychiatric symptoms	Dementia	Dementia, neuropsychiatric	Standard dementia tool	Recall bias
PBA-HD [101]	HD behavioral symptoms	Huntington’s disease	HD	Disease-specific structured ratings	Time-intensive
UHDRS-B NEW [113]	Behavioral snapshot	Huntington’s disease	HD	Brief longitudinal tracking	Single-item aggression
Snaith IS [114]	Irritability	Psychiatry, HD	Psychiatric, neurodegenerative	Brief	Not aggression- specific
IPAS [111]	Aggression style	IED, forensic	Aggressive samples	Impulsive vs. premeditated	Limited regulatory use
LHA [109]	Trait aggression	IED, general	IED research	Enduring aggression trait	Not treatment-sensitive
BPAQ [110]	Trait aggression	General, IED	Clinical/general	Well validated	Self-report bias
OAS-M [115]	Episodic aggression	IED, psychiatric	IED trials	High sensitivity	Incident capture required
MOAS [112]	Aggression incidents	Psychiatric	Inpatient	Weighted severity	Outpatient limits
PANSS-EC [116]	Acute agitation	Schizophrenia	Acute psychosis	Rapid assessment	Agitation not incidents

**Table 4:** Instrument structure and scoring characteristics for aggression-related outcome measures.

Instrument	Administration	Assessment Window	Items/Structure	Scoring	Aggression-Relevant Content
ABC-C [104]	Caregiver-rated	~4 weeks	58 items; 5 subscales	0–3 severity; subscale totals	Aggressive outbursts; self-injury
BPI [106]	Caregiver-rated	Weeks–months	52 items; 3 subscales	Frequency × severity	Hitting, kicking, biting
BEHAVE-AD [99]	Clinician interview	~2 weeks	25 items; 7 clusters	0–3 severity; total score	Physical and verbal aggression
CMAI [98]	Caregiver/staff-rated	Past 2 weeks	29 items; 3 subscales	Frequency 1–7	Physically aggressive agitation
MOAS [112]	Clinician/observer-rated	Past week	4 aggression domains	Weighted score 0–40	Verbal, self-, object-, and person-directed aggression

## Data Availability

No new data were created or analyzed in this study.

## References

[1] Feigin V.L., Vos T., Nichols E., Owolabi M.O., Carroll W.M., Dichgans M., Deuschl G., Parmar P., Brainin M., Murray C. (2020). The global burden of neurological disorders: Translating evidence into policy. Lancet Neurol..

[2] GBD Dementia Forecasting Collaborators (2022). Estimation of the global prevalence of dementia in 2019 and forecasted prevalence in 2050: An analysis for the Global Burden of Disease Study 2019. Lancet Public Health.

[3] Sims J.R., Zimmer J.A., Evans C.D., Lu M., Ardayfio P., Sparks J., Wessels A.M., Shcherbinin S., Wang H., Nery E.S.M. (2023). Donanemab in early symptomatic Alzheimer disease: The TRAILBLAZER-ALZ 2 randomized clinical trial. JAMA.

[4] Van Dyck C.H., Swanson C.J., Aisen P.S., Bateman R.J., Chen C., Gee M., Kanekiyo M., Li D., Reyderman L., Cohen S. (2023). Lecanemab in early Alzheimer’s disease. N. Engl. J. Med..

[5] Miller T., Cudkowicz M., Shaw P.J., Anderson P.M., Atassi N., Bucelli R.C., Genge J., Glass J., Ladha S., Ludoph A.L. (2022). Trial of antisense oligonucleotide tofersen for SOD1 ALS. N. Engl. J. Med..

[6] Schwertner E., Pereira J.B., Xu H., Secnik J., Winblad B., Eriksdotter M., Nägga K., Religa D. (2022). Behavioral and psychological symptoms of dementia in different dementia disorders: A large-scale study of 10,000 individuals. J. Alzheimers Dis..

[7] Gilmore M.C., Stebbins L., Argüelles-Borge S., Trinidad B., Golden C.J. (2020). Development and treatment of aggression in individuals with dementia. Aggress. Violent Behav..

[8] Mok P.L.H., Carr M.J., Guthrie B., Morales D.R., Sheikh A., Elliott R.A., Camacho E.M., van Staa T., Avery A.J., Ashcroft D.M. (2024). Multiple adverse outcomes associated with antipsychotic use in people with dementia: Population-based matched cohort study. Br. Med. J..

[9] Insel T.R., Young L.J. (2010). The challenge of translation in social neuroscience: A review of oxytocin, vasopressin, and affiliative behavior. Neuron.

[10] Marcinkowska A.B., Biancardi V.C., Winklewski P.J. (2022). Arginine vasopressin, synaptic plasticity, and brain networks. Curr. Neuropharmacol..

[11] Rigney N., de Vries G.J., Petrulis A., Young L.J. (2022). Oxytocin, vasopressin, and social behavior: From neural circuits to clinical opportunities. Endocrinology.

[12] Meyer-Lindenberg A., Domes G., Kirsch P., Heinrichs M. (2011). Oxytocin and vasopressin in the human brain: Social neuropeptides for translational medicine. Nat. Rev. Neurosci..

[13] Hu H., Zarate C.A., Verbalis J. (2024). Arginine vasopressin in mood disorders: A potential biomarker of disease pathology and a target for pharmacologic intervention. Psychiatry Clin. Neurosci..

[14] Maibach H.T., Brownstein M.J., Hersch S.M., Anderson K.E., Itzkowitz D.E., Damiano E.M., Simon N.G. (2022). The vasopressin 1a receptor antagonist SRX246 reduces aggressive behavior in Huntington’s disease. J. Pers. Med..

[15] Oliver G., Schafer E.A. (1895). On the physiological action of extracts of pituitary body and certain other glandular organs. J. Physiol..

[16] Howell W.H. (1898). The physiological effects of extracts of the hypophysis cerebri and infundibular body. J. Exp. Med..

[17] Magnus R.S., Schäfer E.A. (1901). Effects of post-pituitary extracts. J. Physiol..

[18] Dale H.H. (1906). On some physiological actions of ergot. J. Physiol..

[19] Ott I., Scott J.C. (1910). The action of infundibulum upon the mammary secretion. Proc. Soc. Exp. Biol. Med..

[20] Kamm O. (1928). The dialysis of pituitary extracts. Science.

[21] du Vigneaud V., Bartlett M.F., Trippett S. (1953). The sequence of amino acids in oxytocin with a proposal for the structure of oxytocin. J. Biol. Chem..

[22] du Vigneaud V., Gish D.T., Katsoyannis P.G. (1954). A synthetic preparation possessing biological properties associated with arginine-vasopressin. J. Am. Chem. Soc..

[23] Acher R., Chauvet J., Olivry G. (1956). Sur l’existence éventuelle d’une hormone unique neurohypophysaire I. Relations entre l’ocytocine, la vasopressine et la protéine de van dyke extraites de la neurohypophyse du boeuf [The actual existence of a single pituitary hormone. I. Relation between the oxytocin, vasopressin, and Van Dyke’s protein, extracted from bovine pituitary]. Biochim. Biophys. Acta.

[24] Gainer H., Sarne Y., Brownstein M.J. (1977). Neurophysin biosynthesis: Conversion of a putative precursor during axonal transport. Science.

[25] Ivell R., Schmale H., Richter D. (1983). Vasopressin and oxytocin precursors as model preprohormones. Neuroendocrinology.

[26] Scharrer E., Scharrer B. (1945). Neurosecretion. Physiol. Rev..

[27] Palkovits M., Brownstein M.J., Arimura A., Sato H., Schally V., Kizer J.S. (1976). Somatostatin content of the hypothalamic ventromedial and arcuate nuclei and circumventricular organs in the rat. Brain Res..

[28] Glick S.M., Brownstein M.J. (1980). Vasopressin content of rat brain. Life Sci..

[29] Weindl A., Sofroniew M.V. (1976). Demonstration of Extrahypothalamic Peptide Secreting Neurons. A Morphologic Contribution to the Investigation of Psychotropic Effects of Neurohormones. Pharmacopsychiatry.

[30] Morel A., O’Carroll A.M., Brownstein M.J., Lolait S.J. (1992). Molecular cloning and expression of a rat V1a arginine vasopressin receptor. Nature.

[31] Kimura T., Tanizawa O., Mori K., Brownstein M.J., Okayama H. (1992). Structure and expression of a human oxytocin receptor. Nature.

[32] Freeman S.M. (2022). Using receptor autoradiography to visualize and quantify oxytocin and vasopressin 1a receptors. Methods Mol. Biol..

[33] Gitelman S.E., Feldman B.J., Rosenthal S.M. (2006). Nephrogenic syndrome of inappropriate antidiuresis: A novel disorder in water balance in pediatric patients. Am. J. Med..

[34] Lolait S.J., O’Carroll A.M., McBride O.W., Konig M., Morel A., Brownstein M.J. (1992). Cloning and characterization of a vasopressin V2 receptor and possible link to nephrogenic diabetes insipidus. Nature.

[35] Thompson R., Gupta S., Miller K., Mills S., Orr S. (2004). The effects of vasopressin on human facial responses related to social communication. Psychoneuroendocrinology.

[36] Zink C.F., Meyer-Lindenberg A. (2012). Human neuroimaging of oxytocin and vasopressin in social cognition. Horm. Behav..

[37] Lee R.J., Coccaro E.F., Cremers H., McCarron R., Lu S.-F., Brownstein M.J., Simon N.G. (2013). A novel V1a receptor antagonist blocks vasopressin-induced changes in the CNS response to emotional stimuli: An fMRI study. Front. Syst. Neurosci..

[38] Lu S.F., Simon N.G., Palkovits M., Brownstein M.J. (1994). Identification and distribution of vasopressin 1a receptor in human and monkey brain. Soc. Neurosci. Abstr..

[39] Young L.J., Toloczko D., Insel T.R. (1999). Localization of vasopressin (V1a) receptor binding and mRNA in the rhesus monkey brain. J. Neuroendocrinol..

[40] Coccaro E.F., Kavoussi R.J., Hauger R.L., Cooper T.B., Ferris C.F. (1998). Cerebrospinal fluid vasopressin levels: Correlates with aggression and serotonin function in personality-disordered subjects. Arch. Gen. Psychiatry.

[41] Zink C.F., Kempf L., Hakimi S., Rainey C.A., Stein J.L., Meyer-Lindenberg A. (2011). Vasopressin modulates social recognition-related activity in the left temporoparietal junction in humans. Transl. Psychiatry.

[42] Zink C.F., Stein J.L., Kempf L., Hakimi S., Meyer-Lindenberg A. (2010). Vasopressin modulates medial prefrontal cortex–amygdala circuitry during Emotion Processing in Humans. J. Neurosci..

[43] Ferris C.F., Stolberg T., Kulkarni P., Murugavel M., Blanchard R., Blanchard D.C., Febo M., Brevard M., Simon N.G. (2008). Imaging the neural circuitry and chemical control of aggressive motivation. BMC Neurosci..

[44] Griebel G., Holsboer F. (2012). Neuropeptide receptor ligands as drugs for psychiatric diseases: The end of the beginning?. Nat. Rev. Drug Discov..

[45] Ring R.H. (2005). The central vasopressinergic system: Examining the opportunities for psychiatric drug development. Curr. Pharm. Des..

[46] Ryckmans T. (2010). Modulation of the vasopressin system for the treatment of CNS diseases. Curr. Opin. Drug Discov. Dev..

[47] Simon N.G., Guillon C., Fabio K., Heindel N.D., Lu S.-F., Miller M., Ferris C.F., Brownstein M.J., Garripa C., Koppel G.A. (2008). Vasopressin antagonists as anxiolytics and antidepressants: Recent Developments. Recent Pat. CNS Drug Discov..

[48] Lago T.R., Brownstein M.J., Page E., Beydler E., Manbeck A., Beale A., Roberts C., Balderston N., Damiano E., Pineles S.L. (2021). The novel vasopressin receptor (V1aR) antagonist SRX246 reduces anxiety in an experimental model in humans: A randomized proof-of-concept study. Psychopharmacology.

[49] Difede J., McAleavey A.A., Emrich M., Jick A., Ovalles A., Wyka K., Spielman L., Olden A., Peskin M., Becket-Davenport C. (2023). A proof-of-concept randomized crossover clinical trial of a first-in-class vasopressin 1a receptor antagonist for PTSD: Design, methods, and recruitment. Contemp. Clin. Trials Commun..

[50] Bolognani F., Del Valle Rubido M., Squassante L., Wandel C., Derks M., Murtagh L., Sevigney J., Khwaja O., Umbricht D., Fontoura P. (2019). A phase 2 clinical trial of a vasopressin V1a receptor antagonist shows improved adaptive behaviors in men with autism spectrum disorder. Sci. Transl. Med..

[51] Meyer-Lindenberg A., Kolachana B., Gold B., Olsh A., Nicodemus K.K., Mattay V., Dean M., Weinberger D.R. (2009). Genetic variants in AVPR1A linked to autism predict amydala activation and personality traits in healthy humans. Mol. Psychiatry.

[52] Hazelton J.L., Carneiro F., Maito M., Richter F., Legaz A., Altschuler F., Cubillos-Pinilla L., Chen Y., Doherty C.P., Baez S. (2025). Neuroimaging meta-analyses reveal convergence of interoception, emotion, and social cognition across neurodegenerative diseases. Biol. Psychiatry.

[53] Koster-Hale J., Richardson H., Velez N., Asaba M., Young L., Saxe R. (2017). Mentalizing regions represent distributed, continuous, and abstract dimensions of others’ beliefs. NeuroImage.

[54] Merchant J.S., Glaros S., Edakoth E., Harris R., Tchangalova N., Redcay E. (2025). Brain bases of real-time social interaction: A meta-analytic investigation of human neuroimaging studies. Apert. Neuro..

[55] Ahmad N., Zorns S., Chavarria K., Brenya J., Janowska A., Keenan J.P. (2021). Are We Right about the Right TPJ? A Review of Brain Stimulation and Social Cognition in the Right Temporal Parietal Junction. Symmetry.

[56] Rosenberg P.B., Nowrangi M.A., Lyketsos C.G. (2015). Neuropsychiatric symptoms in Alzheimer’s disease: What might be associated brain circuits?. Mol. Asp. Med..

[57] Lattanzio L., Seames A., Holden S.K., Buard I. (2021). The emergent relationship between temporoparietal junction and anosognosia in Alzheimer’s disease. J. Neurosci. Res..

[58] Peng S., Schaper F.L.W.V.J., Cohen-Zimerman S., Miller G.N., Jiang J., Rouhl R.P.W., Temel Y., Siddiqi S.H., Grafman J., Fox M.D. (2025). Mapping lesion-related human aggression to a common brain network. Biol. Psychiatry.

[59] da Cunha-Bang S., Fisher P.M., Hjordt L.V., Perfalk E., Skibsted A.P., Bock C., Baandrup A.O., Deen M., Thomsen C., Sestoft D.M. (2017). Violent offenders respond to provocations with high amygdala and striatal reactivity. Soc. Cogn. Affect. Neurosci..

[60] Siep N., Tonnaer F., van de Ven V., Arntz A., Raine A., Cima M. (2019). Anger provocation increases limbic and decreases medial prefrontal cortex connectivity with the left amygdala in reactive aggressive violent offenders. Brain Imaging Behav..

[61] Loy J.H., Merry S.N., Hetrick S.E., Stasiak K. (2017). Atypical antipsychotics for disruptive behaviour disorders in children and youths. Cochrane Database Syst. Rev..

[62] Meza N., Franco J.V., Sguassero Y., Nunez V., Liquitay C.M.E., Rees R., Williams K., Rojas V., Royas R., Pringsheim T. (2025). Atypical antipsychotics for autism spectrum disorder: A network meta-analysis. Cochrane Database Syst. Rev..

[63] Serradeil-Le Gal C., Wagnon J., Valette G., Garcia G., Pasacal M., Maffrand J.P., Le Fur G. (2002). Nonpeptide vasopressin receptor antagonists: Development of selective and orally active V1a, V2 and V1b receptor ligands. Prog. Brain Res..

[64] Jacob S., Anagnostou E., Hollander E., Jou R., McNamara N., Sikich L., Tobe R., Murphy D., McCracken N., Ashford E. (2022). Large multicenter randomized trials in autism: Key insights gained from balovaptan clinical development program. Mol. Autism.

[65] Hollander E., Jacob S., Jou R., McNamara N., Sikich L., Tobe R., Smith J., Sanders K., Squassante L., Murtagh L. (2022). Balovaptan vs. placebo for social communication in childhood autism spectrum disorder. JAMA Psychiatry.

[66] Fisher C.A., Sewell K., Brown A., Churchyard A. (2014). Aggression in Huntington’s disease: A systematic review of rates of aggression and treatment methods. J. Huntington’s Dis..

[67] van Duijn E., Kingma E.M., van der Mast R.C. (2007). Psychopathology in verified Huntington’s disease gene carriers. J. Neuropsychiatry Clin. Neurosci..

[68] Olvera C., Stebbins G.T., Patron Romero V., Hall D.A. (2022). Criminality in Huntington disease. Neurol. Clin. Pract..

[69] Ruiz-Idiago J., Pomarol-Clotet E., Salvador R. (2023). Longitudinal analysis of neuropsychiatric symptoms in a large cohort of early-moderate manifest Huntington’s disease patients. Parkinsonism Relat. Disord..

[70] Eaton J.E., Claassen D.O. (2024). Guidance on antipsychotic selection for agitation and aggressive behavior in persons with Huntington’s disease. Expert Rev. Neurother..

[71] Brownstein M.J., Simon N.G., Long J.D., Yankey J., Maibach H., Cudkowicz M., Coffey C., Conwit R.A., Lungu C., Anderson C. (2020). Safety and tolerability of SRX246, a vasopressin 1a antagonist, in irritable Huntington’s disease patients—A randomized phase 2 clinical trial. J. Clin. Med..

[72] Coccaro E.F., Lee R.J., Kavoussi R.J. (2009). A double-blind, randomized, placebo-controlled trial of fluoxetine in patients with intermittent explosive disorder. J. Clin. Psychiatry.

[73] Silva H., Iturra P., Solari A., Villarroel J., Jerez S., Jimenez M., Galleguillos F., Bustamante M.L. (2010). Fluoxetine response in impulsive–aggressive behavior and serotonin transporter polymorphism in personality disorder. Psychiatr. Genet..

[74] Hollander E., Tracy K.A., Swann A.C., Coccaro E.F., McElroy S.L., Wozniak P., Sommerville K.W., Nemeroff C.B. (2003). Divalproex in the Treatment of Impulsive Aggression: Efficacy in Cluster B Personality Disorders. Neuropsychopharmacology.

[75] Mattes J.A. (2005). Oxcarbazepine in patients with impulsive aggression: A double-blind, placebo-controlled trial. J. Clin. Psychopharmacol..

[76] Mattes J.A. (2008). Levetiracetam in patients with impulsive aggression: A double-blind, placebo-controlled trial. J. Clin. Psychiatry.

[77] Côté V., Knoth I.S., Lalancette È. (2020). ehavioural Characteristics Related to Adaptive Functioning in Young Persons with Tuberous Sclerosis Complex, Down Syndrome and Fragile x Syndrome. J. Dev. Phys. Disabil..

[78] Crawford H., Karakatsani E., Singla G., Oliver C. (2019). The persistence of self-injurious and aggressive behavior in males with fragile X syndrome over 8 years: A longitudinal study of prevalence and predictive risk markers. J. Autism Dev. Disord..

[79] Eckert E.M., Dominick K.C., Pedapati E.V., Wink L.K., Shaffer R.C., Andrews H., Choo T.H., Chen C., Kaufmann W.E., Tartaglia N. (2019). Pharmacologic interventions for irritability, aggression, agitation and self-injurious behavior in fragile X syndrome: An Initial Cross-Sectional Analysis. J. Autism Dev. Disord..

[80] Berry-Kravis E., Potanos K. (2004). Psychopharmacology in fragile X syndrome—Present and future. Ment. Retard. Dev. Disabil. Res. Rev..

[81] Berry-Kravis E., Sumis A., Hervey C., Mathur S. (2012). Clinic-Based Retrospective Analysis of Psychopharmacology for behavior in fragile X syndrome. Int. J. Pediatr..

[82] Valdovinos M.G., Epperson C., Johnson C. (2023). A review of the use of psychotropic medication to address challenging behaviour in neurodevelopmental disorders. Int. Rev. Neurobiol..

[83] Deb S., Unwin G. (2007). Psychotropic medication for behaviour problems in people with intellectual disability: A review of the current literature. Curr. Opin. Psychiatry.

[84] Berry-Kravis E., Sumis A., Hervey C., Nelson M., Porges S.W., Weng N., Weiler I.J., Greenough W.T. (2008). Open-label treatment trial of lithium to target the underlying defect in fragile X syndrome. J. Dev. Behav. Pediatr..

[85] Tsiouris J.A., Brown W.T. (2004). Neuropsychiatric symptoms of fragile X syndrome. CNS Drugs.

[86] Eronen M., Angermeyer M.C., Schulze B. (1998). The psychiatric epidemiology of violent behaviour. Soc. Psychiatry Psychiatr. Epidemiol..

[87] Neumann I.D., Veenema A.H., Beiderbeck D.I. (2010). Aggression and anxiety: Social context and neurobiological links. Front. Behav. Neurosci..

[88] Davidson J.R. (2000). Social anxiety disorder under scrutiny. Depress. Anxiety.

[89] Moscovitch D.A., McCabe R.E., Antony M.M., Rocca L., Swinson R.P. (2008). Anger experience and expression across the anxiety disorders. Depress. Anxiety.

[90] Kashdan T.B., McKnight P.E. (2010). The darker side of social anxiety. Curr. Dir. Psychol. Sci..

[91] Breen W.E., Kashdan T.B. (2011). Anger suppression after imagined rejection among individuals with social anxiety. J. Anxiety Disord..

[92] Galbraith T., Heimberg R.G., Wang S., Scheier F.R., Blanco C. (2014). Comorbidity of social anxiety disorder and antisocial personality disorder in the National Epidemiological Survey on Alcohol and Related Conditions (NESARC). J. Anxiety Disord..

[93] Keyes K.M., McLaughlin K.A., Vo T., Galbraith T., Heimberg R.G. (2016). Anxious and aggressive: The co-occurrence of IED with anxiety disorders. Depress. Anxiety.

[94] Fava M. (1998). Depression with anger attacks. J. Clin. Psychiatry.

[95] Mammen O.K., Kolko D.J., Pilkonis P.A. (2002). Negative affect and parental aggression in child physical abuse. Child Abuse Negl..

[96] Liu Q., Cole D.A. (2021). Aggressive outbursts among adults with major depressive disorder: Results from the Collaborative Psychiatric Epidemiological Surveys. J. Psychiatr. Res..

[97] Cummings J.L., Mega M., Gray K., Rosenberg-Thompson S., Carussi D.A., Gornbein J. (1994). The Neuropsychiatric Inventory: Comprehensive assessment of psychopathology in dementia. Neurology.

[98] Cohen-Mansfield J. (1986). Agitated behaviors in the elderly: I. A conceptual review. J. Am. Geriatr. Soc..

[99] Reisberg B., Borenstein J., Salob S.P., Ferris S.H., Franssen E., Georgotas A. (1987). Behavioral symptoms in Alzheimer’s disease: Phenomenology and treatment. J. Clin. Psychiatry.

[100] Hallikainen I., Hongisto K., Välimäki T., Hanninen T., Martikainen J., Koivisto A.M. (2018). The progression of neuropsychiatric symptoms in Alzheimer’s disease during a five-year follow-up: Kuopio ALSOVA study. J. Alzheimer’s Dis..

[101] Craufurd D., Thompson J.C., Snowden J.S. (2001). Behavioral changes in Huntington disease. Neuropsychiatry Neuropsychol. Behav. Neurol..

[102] Thompson J.C., Harris J., Sollom A.C., Stopford C.L., Howard E., Snowden J.S., Craufurd D. (2012). Longitudinal evaluation of neuropsychiatric symptoms in Huntington’s disease. J. Neuropsychiatry Clin. Neurosci..

[103] Callaghan J., Stopford C., Arran N., Boisse M.-F., Coleman A., Santos R.D., Dumas E.M., Hart E.P., Justo D., Owen G. (2015). Reliability and Factor Structure of the Short Problem Behaviors Assessment for Huntington’s Disease (PBA-s) in the TRACK-HD and REGISTRY studies. J. Neuropsychiatry.

[104] Aman M.G., Singh N.N., Stewart A.W., Field C.J. (1985). The Aberrant Behavior Checklist: A behavior rating scale for the assessment of treatment effects. Am. J. Ment. Defic..

[105] Sansone S.M., Widaman K.F., Hall S.S., Reiss A.L., Lightbody A., Kaufmann W.E., Berry-Kravis E., Lachiewicz A., Brown E.C., Hessl D. (2012). Psychometric study of the Aberrant Behavior Checklist in fragile X syndrome and implication for targeted treatment. J. Autism Dev. Disord..

[106] Rojahn J., Matson J.L., Lott D., Esbensen A.J., Smalls Y. (2001). The Behavior Problems Inventory: An instrument for the assessment of self-injury, stereotyped behavior, and aggression/destruction in individuals with developmental disabilities. J. Autism Dev. Disord..

[107] Yudofsky S.C., Silver J.M., Jackson W., Endicott J., Williams D. (1986). The Overt Aggression Scale for the objective rating of verbal and physical aggression. Am. J. Psychiatry.

[108] Silver J.M., Yudofsky S.C., Anderson K.E. (1991). Modified Overt Aggression Scale. J. Neuropsychiatry Clin. Neurosci..

[109] Coccaro E.F., Berman M.E., Kavoussi R.J. (1997). Assessment of life history of aggression: Development and psychometric characteristics. J. Psychiatr. Res..

[110] Buss A.H., Perry M. (1992). The aggression questionnaire. J. Pers. Soc. Psychol..

[111] Stanford M.S., Houston R.J., Mathias C.W., Villemarette-Pittman N.R., Helfritz L.E., Conklin S.M. (2003). Characterizing aggressive behavior. Assessment.

[112] Patel V., Hope R.A. (1992). A rating scale for aggressive behaviour in the elderly—The RAGE. Psychol. Med..

[113] (1996). Unified Huntington’s Disease Rating Scale: Reliability and consistency. Huntington Study Group. Mov. Disord..

[114] Snaith R.P., Taylor C.M. (1985). Irritability: Definition, assessment and associated factors. Br. J. Psychiatry.

[115] Coccaro E.F. (2020). The Overt Aggression Scale Modified (OAS-M) for clinical trials targeting impulsive aggression and intermittent explosive disorder: Validity, reliability, and correlates. J. Psychiatr. Res..

[116] Montoya A., Valladares A., Lizán L., San L., Escobar R., Paz S. (2011). Validation of the Excited Component of the Positive and Negative Syndrome Scale (PANSS-EC) in a naturalistic sample of 278 patients with acute psychosis and agitation in a psychiatric emergency room. Health Qual Life Outcomes.

[117] Kay S.R., Wolkenfeld F., Murrill L.M. (1988). Profiles of aggression among psychiatric patients. J. Nerv. Ment. Dis..

[118] Beher J., Treml E., Wintle B. (2025). Group discussions improve reliability and validity of rated categories based on qualitative data from systematic review. PLoS ONE.

